# C-DTW for Human Action Recognition Based on Nanogenerator

**DOI:** 10.3390/s23167230

**Published:** 2023-08-17

**Authors:** Haifeng Xu, Renhai Feng, Weikang Zhang

**Affiliations:** School of Electrical and Information Engineering, Tianjin University, Tianjin 300072, China; xuhaifeng@tju.edu.cn (H.X.); zhangweikang@tju.edu.cn (W.Z.)

**Keywords:** dynamic time warping, distance-based time series classification, human action recognition, nanogenerator

## Abstract

Sensor-based human action recognition (HAR) is considered to have broad practical prospects. It applies to wearable devices to collect plantar pressure or acceleration information at human joints during human actions, thereby identifying human motion patterns. Existing related works have mainly focused on improving recognition accuracy, and have rarely considered energy-efficient management of portable HAR systems. Considering the high sensitivity and energy harvesting ability of triboelectric nanogenerators (TENGs), in this research a TENG which achieved output performance of 9.98 mW/cm${}^{2}$ was fabricated using polydimethylsiloxane and carbon nanotube film for sensor-based HAR as a wearable sensor. Considering real-time identification, data are acquired using a sliding window approach. However, the classification accuracy is challenged by quasi-periodic characteristics of the intercepted sequence. To solve this problem, compensatory dynamic time warping (C-DTW) is proposed, which adjusts the DTW result based on the proportion of points separated by small distances under DTW alignment. Our simulation results show that the classification accuracy of C-DTW is higher than that of DTW and its improved versions (e.g., WDTW, DDTW and softDTW), with almost the same complexity. Moreover, C-DTW is much faster than shapeDTW under the same classification accuracy. Without loss of generality, the performance of the existing DTW versions can be enhanced using the compensatory mechanism of C-DTW.

## 1. Introduction

Human action recognition (HAR) refers to the recognition of various human activities through signal processing. It is widely applied in competitive sports, health detection, medical research, and pedestrian navigation [1]. HAR is an emerging application direction in human–computer interaction, such as using gestures to control computers or robots and providing real-time feedback on human actions to virtual environments, allowing users to have a better virtual reality experience [2]. Research on HAR can be categorized into video-based HAR and sensor-based HAR. Video-based HAR is primarily used to analyze human behavior in videos and conduct real-time monitoring [3], in which multispectral devices, cameras, infrared spectrometers, etc., are used to collect video or image data. The analysis of these data necessitates careful consideration of the instrument’s position as well as the influence of various factors, including lighting, occlusion, background, and object angle. These factors pose challenges when attempting to employ video-based HAR. Furthermore, the high cost of video recording equipment and the significant computing resources needed for video recognition algorithms such as Convolutional Neural Network [4] and Vision Transformer [5] have restricted the widespread adoption of this technology among individuals.

Sensor-based HAR utilizes data on human activity and the environment collected through sensor devices, such as accelerometers, gyroscopes, and magnetometers. Consequently, sensor-based HAR offers several advantages, including low cost, convenience, noninvasiveness, and enhanced privacy protection, by leveraging sensors to gather the necessary data. In addition, sensor technology has developed in various aspects over the past decade, including computing power, size, accuracy, and manufacturing cost. Nowadays, sensor devices can be readily integrated into portable devices such as mobile phones and watches as well as larger devices such as cars, walls, and furniture. As a result, there is growing interest in wearable sensor-based Human Activity Recognition [6]. Sensor data are collected in natural chronological order with a specific time interval, and comprise real-valued measurements; these are categorized as time series data [7]. Consequently, the sensor-based human action recognition problem primarily revolves around the classification of time-series data. In [8], environmental sensors were used to implement HAR tasks in multi-tenant smart home scenarios. In [9], the authors focused on the accurate classification of daily human activities from accelerometer and gyroscope sensor data. To reduce the computational time and storage of data processing, ref. [10] proposed a semisupervised method called tri-very fast decision tree, which was applied to embedded devices after applying the simulated annealing algorithm for feature selection. In order to identify complex actions, ref. [11] took into consideration the correlation between different sensor positions on the body, achieving high action recognition accuracy and generalization ability. From the above studies, it is apparent that using new types of sensors and developing action recognition algorithms based on these new sensors is an interesting problem in the field of sensor-based HAR. Consequently, we propose the use of a contact–separation TENG as a sensor for HAR tasks. When the positive and negative electrodes of the TENG are connected to the load, the voltage waveform of the load becomes associated with the movement of the nanogenerator’s electrodes. By establishing a correlation between human body movements and the movement of the nanogenerator’s electrodes, the HAR task can be accomplished effectively. Moreover, a sensor based on TENG and a piezoelectric nanogenerator (PENG) has the advantages of low power consumption, high sensitivity, high stability, and low cost [12].

Since [13] first fabricated TENGs, achieving 10.4 mW/cm${}^{2}$ output performance, research on nanogenerators have made breakthroughs in terms of power generation efficiency, sensitivity, and production processes. Nanogenerators, including PENG and TENG varieties, can effectively convert mechanical energy into electrical power or signals [14]. PENGs exhibit the advantage of providing a stable output that remains unaffected by environmental factors such as temperature and humidity. However, their application is limited due to the constrained piezoelectric and mechanical properties of the piezoelectric nanowires or composite films that they use, restricting their ability to harvest energy to only a specific range of weak mechanical energy [15]. Thus, we did not select a PENG for this paper. In recent years, TENGs have seen significant advancements in terms of their material structure and functionality. To achieve a more stable power supply, ref. [16] developed a fully stretchable and highly durable one-horsepower TENG with gold nanosheets embedded into both a PDMS matrix and a micropyramid-patterned PDMS. By introducing 4D printing technology to manufacture a transparent self-recovery TENG using a fused deposition modeling (FDM) printer and spray technique, ref. [17] achieved excellent self-recovery capability of device performance, improved the robustness of the device structure, and achieved an output performance of 56 mW/m${}^{2}$. In [18], fragile and rigid mica with strong triboelectric positivity was exfoliated into 2D nanosheets and electrospun into flexible and stretchable thermoplastic polyurethane nanofibers for a high-performance TENG that achieved 1458 mW/m${}^{2}$ output performance. Although TENGs have seen major breakthroughs in energy harvesting ability, and potential applications are becoming widespread, there are three difficulties when applying them to a wearable sensor-based HAR(ws-HAR) system:

(i) The contradiction between large-scale production and high-performance products has not been resolved;
(ii) The nonlinear relationship between pressure and generated voltage makes it difficult to directly infer pressure magnitude through voltage;
(iii) Energy harvesting remains challenging, as low-frequency components account for a large proportion of the generated energy.

Fortunately, the time-domain voltage waveform of TENGs has specific characteristics which can be utilized in WS-HAR. WS-HAR can be treated as a typical time series classification task. Common classifiers for times series include support vector machine [19], deep belief network [20], convolutional network [21,22], long short-term memory network [23], and transformer [24]. Deep learning methods often suffer from long training inference times, which hinders their advantage to handle real-time processing demands in the context of WS-HAR. Moreover, support vector machine heavily relies on heuristic hand-crafted feature extraction before classification. Unlike the aforementioned classification methods, DTW has the advantage of invariance against signal warping (shifting and scaling in time), leading to its becoming one of the most preferable measures in classification tasks for time series data [25].

In order to address practical applications, extensive research was conducted on DTW. To improve path searching speed, ref. [26] assumed a limited optimal warping path range and proposed an adaptive window restriction method. In order to achieve more reasonable matching, ref. [27] weighted the distance matrix. In [28], the sequence was differentiated before DTW to make the alignment more rational. In [29], the authors improved DTW alignment by considering the local structural information of the sequence. In order to use DTW as the loss function of neural network, ref. [30] applied a soft minimum. Subsequently, DTW has been applied in machine learning as a loss function for times series classification tasks such as HAR [31,32] and EEG classification [33]. The majority of the current research concentrates on integrating DTW with other algorithms to explore its application in new scenarios and to suppress cases of one-to-many DTW in order to achieve improved shape-based point-to-point correspondences. One aspect that has often been overlooked is the selection of DTW alignment. Therefore, in this paper we propose a compensatory mechanism based on correspondence selection to address the aforementioned limitations. Moreover, this compensatory mechanism enables quasi-periodic sequences to attain satisfactory classification accuracy without requiring endpoint detection.

Considering all the facts mentioned above, this paper proposes a recognition framework based on TENG and DTW with a compensatory mechanism. The main contributions of this paper are listed as follows:

- Application of new materials: carbon nanotubes (CNTs) doped in PDMS were transferred to commercial silver textiles using a novel brush method to fabricate the TENG and the application feasibility of the TENG was proven through experiments. This solves contradiction (i) above.
- Flexible compensatory mechanism: the compensatory mechanism based on DTW alignment selection proposed in this paper can be flexibly applied to improved DTW. The compensatory mechanism can increase the weighting of information from alignments with similar shapes while reducing the weighting of information from alignments with dissimilar shapes caused by greedy algorithm. Moreover, as the improved DTW focuses on alignment based on the shape of the sequence, the compensatory mechanism can achieve a greater performance improvement.
- Improvement of DTW: under the traditional DTW framework, misclassification occurs when a sequence exhibits incompleteness after interception. Therefore, compensatory DTW (C-DTW) is proposed to solve this deficiency. The DTW result is compensated using the proportion of points with small distance under DTW alignment, making C-DTW more robust against quasi-periodic sequence classification, thereby solving contradiction (ii) in WS-HAR.
- Simplification of WS-HAR system: the combination of energy harvesting and sensing through TENG can simplify the system design. Moreover, neither endpoint detection nor manual feature extraction are required when using C-DTW. As a result, the proposed WS-HAR with TENG approach can be more efficient than traditional HAR system, opening up the possibility of eventually solving contradiction (iii).

In this paper, we use a specialized TENG to collect mechanical energy. The TENG possesses high performance and is a good fit for mass production. The sensor is able to realize human action recognition based on voltage waveforms. The rest of this paper is organized as follows. In Section 2, we demonstrate how to accommodate TENGs in WS-HAR and propose a fabrication method. Section 3 describes traditional DTW and the proposed C-DTW. In Section 4, the WS-HAR block diagram is provided and the performance of the C-DTW approach is verified through experiments. In Section 5, the conclusion of this paper is provided.

## 2. HAR Sensor

### 2.1. Theoretical Analysis of TENG Sensor

**Figure 1:**
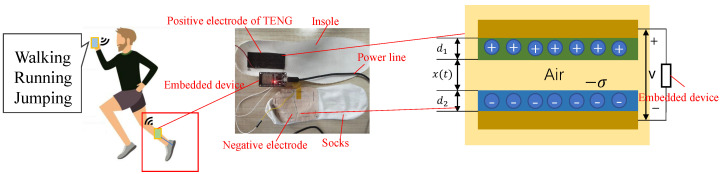
A TENG is placed at the heel and connected with a voltage detection device (or small voltage detection embedded device); the schematic diagram of the TENG is shown on the right.

TENGs can convert mechanical energy into electrical energy through the coupling effect between friction induction and electrostatic induction. To facilitate the analysis of sensor properties in contact-mode TENGs, two dielectric plates are stacked face to face as two triboelectric layers, as shown in Figure 1. The distance x(t) between the two triboelectric layers can be varied under external force, i.e., plantar pressure in this paper. As a result, there are three states in the power generation process of TENGs: (1) when x(t)=0, two contact surfaces form charges with opposite polarity; (2) after contact, i.e., x(t)≠0, the triboelectric layers have opposite static charges with equal density of σ and form an induced potential difference between two metal electrodes. Differences in the pressure placed on the TENG lead to different microcontact areas between the two triboelectric layers, meaning that σ varies with the pressurel (3) when x(t)=0 again, the potential difference formed by the friction charge disappears and the electrons return. For one working cycle of TENG, σ is constant. If two metal electrodes are connected by resistor *R*, electrons flow from one electrode to the other through *R*. According to [34], the V−Q−x relationship of a contact-mode TENG can be written as follows:

(1) $$V\left(t\right)=-\frac{Q(t)}{S\varepsilon_{0}}\left(\frac{d_{1}}{\varepsilon_{1}}+\frac{d_{2}}{\varepsilon_{2}}+x\left(t\right)\right)+\frac{\sigma x(t)}{\varepsilon_{0}}$$

where V(t) is voltage between the two electrodes, Q(t) is the amount of transferred charge, $\varepsilon_{0}$ is the permittivity of air, $\varepsilon_{1}$ and $\varepsilon_{2}$ are the relative permittivities of the triboelectric material, and *S* is the area size of the metals.

When the contact-mode TENG is connected to the resistor *R*, the voltage generated by the internal electric field of the TENG (the right side in (2)) is equal to the voltage of external circuit (the left side in (2)).

(2) $$R\frac{dQ(t)}{t}=-\frac{Q(t)}{S\varepsilon_{0}}\left(\frac{d_{1}}{\varepsilon_{1}}+\frac{d_{2}}{\varepsilon_{2}}+x\left(t\right)\right)+\frac{\sigma x(t)}{\varepsilon_{0}}$$

Assuming that there is no charge transfer between the two electrodes at t=0, we obtain

(3) $${Q\left(t\right)|}_{t=0}=0.$$

Assuming

(4) $$c_{0}=\frac{d_{1}}{\varepsilon_{1}}+\frac{d_{2}}{\varepsilon_{2}},$$

the solution of (2) can be as follows:

(5) $$\begin{array}{cc} Q(t)= & \sigma S-\sigma S\exp\left[-\frac{1}{RS\varepsilon_{0}}\left(c_{0}t+\int_{0}^{t}x\left(t\right)dt\right)\right]\\ \; & -\frac{\sigma c_{0}}{R\varepsilon_{0}}\exp\left[-\frac{1}{RS\varepsilon_{0}}\left(c_{0}t+\int_{0}^{t}x\left(t\right)dt\right)\right]\\ \; & \times \int_{0}^{t}\exp\left[\frac{1}{RS\varepsilon_{0}}\left(c_{0}z+\int_{0}^{z}x\left(z\right)dz\right)\right]dz. \end{array}$$

Combining the definition of the current with Ohm’s law, the output voltage of the TENG is

(6) $$\begin{array}{cc} V(t)= & RI\left(t\right)=R\frac{dQ(t)}{dt}. \end{array}$$

When the foot steps on the TENG, the pressure differential caused by various actions means that the two materials have different degrees of friction; this directly affects σ in (2), which in turn causes V(t) to change. Different actions lead to variations in the regularity of x(t), directly affecting V(t) according to (7). In this way, TENGs can be used in WS-HAR. An embedded voltage detection device can store V(t) with a sampling interval of δt. Assuming that the time frame is t=1,…,I, the voltage sequence can be expressed by the following equation.

(7) $$\begin{array}{c} v=\left[V\left(1\right),\ldots ,V\left(t\right),\ldots ,V\left(I\right)\right]={\left[v_{i}\right]}_{1\times I} \end{array}$$

### 2.2. TENG Preparation

Based on the above theoretical analysis of TENGs and following [35], a more specific TENG design for WS-HAR and mass production is proposed below and its parameters are optimized. The manufacturing process of the TENG for WS-HAR is threefold. First, a 3 cm × 3 cm silver textile is soaked in anhydrous ethanol. Second, CNTs are added to the curing agent and the mixture is combined with PDMS in a mass ratio of 1:10 and remixed for 10 min. Third, 1 g of the mixture solution is transferred by brush to the commercial silver textile. Compared with the traditional dip-coating method, which forms a flat and smooth surface, the brush method used in this paper can form a wavy shape on the macro-level; this can method can be used in large-scale manufacturing. Finally, the silver textile is placed in a vacuum dryer for 20 min, then transferred to an oven with the temperature adjusted to 75 ${}^{\circ }$C for 1.5 h.

## 3. HAR Method

### 3.1. Traditional DTW

Human beings have specialized action habits which do not change over the short term [36]; in this case, the plantar pressure regularity and the separation distance between the heel and insole remain almost constant. When the TENG is placed at the heel, its output voltage waveform (V(t) in (2)) is related to foot movements, which influence x(t) and σ in (2). DTW is a robust method for measuring the similarity between two time series through norm distance [37]; moreover, DTW is applicable to both univariate time series and multivariate time series [35]. In this paper, DTW is combined with 1-NN, which uses local memory as a template. As a result, the feature extraction steps of HAR can be avoided via DTW during recognition.

Suppose that the sequences $\hat{v}={\left[{\hat{v}}_{m}\right]}_{1\times M}$ and $\tilde{v}={\left[{\tilde{v}}_{n}\right]}_{1\times N}$ represent two different voltage sequences; in order to align $\hat{v}$ and $\tilde{v}$, the distance matrix $D\in R^{M\times N}$ is constructed by calculating the distance between each point in $\hat{v}$ and $\tilde{v}$. Element $d_{m,n}$ in D is calculated by

(8) $$d_{m,n}=\sqrt{{\left({\hat{v}}_{m}-{\tilde{v}}_{n}\right)}^{2}}.$$

The warping path is calculated between adjacent points in D according to the following criteria: the DTW warping paths of $\hat{v}$ and $\tilde{v}$ are expressed as a set $W=\{w_{1},\ldots ,w_{t},\ldots w_{T}\}$ with max(M,N)≤T<M+N−1, where $w_{t}=\left(m,n\right)$ indicates that ${\hat{v}}_{m}$ matches ${\tilde{v}}_{n}$. Each element of W needs to meet the following conditions:

(1) Boundedness
  (9) $$w_{1}=\left(1,1\right),w_{T}=\left(M,N\right)$$
(2) Continuity
  (10) $$m-m^{{}^{\prime }}\leq 1,n-n^{{}^{\prime }}\leq 1$$
(3) Monotonicity
  (11) $$m-m^{{}^{\prime }}\geq 0,n-n^{{}^{\prime }}\geq 0,$$

where $m^{{}^{\prime }}$ and $n^{{}^{\prime }}$ are the elements of $w_{t-1}$. According to (9)–(11), we can find that $\left(m^{{}^{\prime }}+1,n^{{}^{\prime }}\right)$, $\left(m^{{}^{\prime }},n^{{}^{\prime }}+1\right)$ or that $(m^{{}^{\prime }}+1,n^{{}^{\prime }}+1)\in W$.

Greedy dynamic programming is utilized to calculate the warping path in D [38]; specifically,

(12) $$\mathrm{DTW}\left(1,1\right)=d_{1,1},$$

(13) $$\mathrm{DTW}\left(m,n\right)=d_{m,n}+\min\left\{\begin{array}{c} \mathrm{DTW}(m,n-1),\\ \mathrm{DTW}(m-1,n-1),\\ \mathrm{DTW}(m-1,n). \end{array}\right\}$$

**Figure 2:**
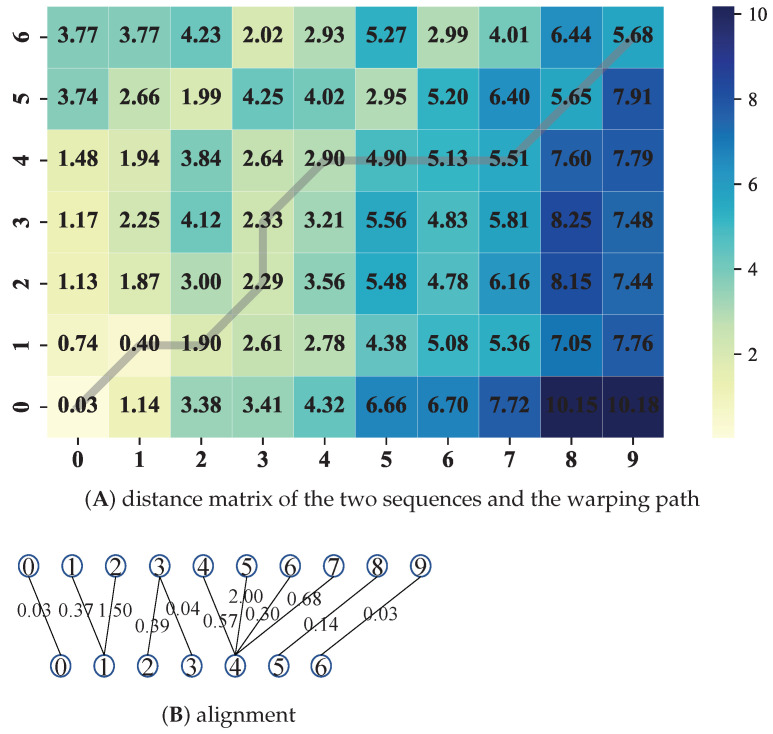
(**A**) The distance matrix and warping path; each element of the distance matrix represents the Euclidean distance between corresponding points of the two sequences. (**B**) The alignment of the example sequences $\hat{v}=\left[-0.95,-0.21,-1.31,-0.96,-0.61,1.34,-0.95\right]$ and $\tilde{v}=\left[-0.92,0.16,1.29,-0.92,-0.04,1.39,-0.91,0.07,1.48,-0.92\right]$; the numbers along the lines represent the Euclidean distances between aligned points, while the numbers inside the circles represent the indices of the example sequences.

The DTW algorithm is presented in Algorithm 1. The distance matrix $D\in R^{7\times 10}$ for the two sequences $\hat{v}=[-0.95,-0.21,-1.31,-0.96,$−0.61,1.34,−0.95] and $\tilde{v}=\left[-0.92,0.16,1.29,-0.92,-0.04,1.39,-0.91,0.07,1.48,-0.92\right]$ is shown in Figure 2A, while the alignment of these two sequence is shown in Figure 2B.
**Algorithm 1** DTW**Require:**sequence $\hat{v}={\left[{\hat{v}}_{m}\right]}_{1\times M}$sequence $\tilde{v}={\left[{\tilde{v}}_{n}\right]}_{1\times N}$**Ensure:**the DTW distance between $\hat{v}$ and $\tilde{v}$the optimal warping path: W**initialize:***M* = length($\hat{v}$)*N* = length($\tilde{v}$)$\mathrm{DTW}\left(1,1\right)=d_{1,1}$**for** m=1 to *M* **do**   **for** n=1 to *N* **do**     $d_{m,n}=\sqrt{{\left({\hat{v}}_{m}-{\tilde{v}}_{n}\right)}^{2}}$;     execute (13) to obtain DTW(m,n) and $w_{t}\in W$   **end for****end for****return:** DTW(M,N), W;

### 3.2. Compensatory DTW

DTW is usually combined with K-nearest neighbor (KNN) for classification; thus, the shapes between the two sequences match. When K=1 in KNN, this method is denoted as DTW+1NN. To best of our knowledge, the predominant focus of current research lies in integrating DTW with other algorithms [31,32,33] in order to investigate its applicability in novel scenarios. Another research focus lies in tackling the challenge of one-to-many alignment issues in order to achieve enhanced shape-based point-to-point alignments. However, researchers have not taken into account that there are similar parts shared among the shapes of the same class of sequences in time-series classification tasks using DTW. When aligned by DTW, these similar parts exhibit smaller distances than dissimilar parts. Therefore, a compensatory mechanism based on DTW alignment selection using these distance properties is proposed here. The proposed compensatory mechanism offers the further advantage of facilitating quasi-periodic sequences to attain satisfactory classification accuracy without requiring endpoint detection. The proposed compensatory DTW (C-DTW) is able to solve the above deficiencies.

**Figure 3:**
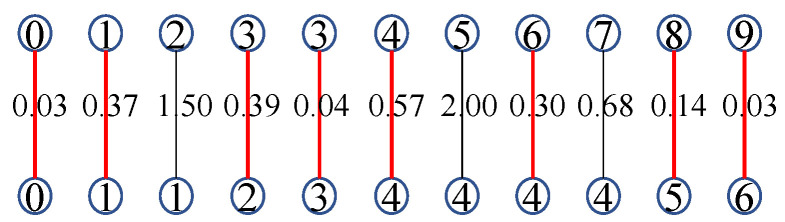
Unfolding the one-to-many alignment; the alignments that satisfy (14) are highlighted by the red lines.

For a point $w_{t}=\left(m,n\right)$ in warping path W, a maximum tolerance α is determined; when the distance between ${\hat{v}}_{m}$ and ${\tilde{v}}_{n}$ satisfies

(14) $$d_{m,n}<\alpha ,$$

then (m,n) is input to the optional shape indicator, i.e., (m,n)∈S. From this, it is easy to draw the conclusion that the probability of two sequences indicating the same actions becomes higher when there are more points in S. Assuming that $N_{\hat{v}}$ and $N_{\tilde{v}}$ are the number of non-repeating indices in $\hat{v}$ and $\tilde{v}$ which are input to S, as shown in Figure 3, the number of subscripts in the red part should satisfy (14). The compensation coefficient $\gamma_{c}$ is defined as

(15) $$\gamma_{c}=1-\frac{\min(N_{\hat{v}},N_{\tilde{v}})}{(M+N)/2};$$

thus, $C-\mathrm{DTW}\left(M,N\right)=\mathrm{DTW}\left(M,N\right)\times \gamma_{c}$. For example, based on the DTW alignment between the example sequences $\hat{v}$ and $\tilde{v}$, we can replicate the one-to-many points issue to calculate the DTW distance in Euclidean space, as shown in Figure 3. Assuming that α=0.6, according to (14) the selected alignment is indicated by the red lines; the number of non-repeating indices $N_{\hat{v}}=6$ with (0, 1, 2, 3, 4, 5, 6) and $N_{\tilde{v}}$ with (0, 1, 3, 4, 6, 8, 9). Using Equation (15), we can obtain the value of $\gamma_{c}$ as $1-\frac{6}{(10+7)/2}=0.294$. Thus, the value of C-DTW is 5.68×0.294=1.67.

In this paper, because the original data are intercepted by the sliding window, the length of two sequences is equal, i.e., M=N. Thus, (15) can be rewritten as

(16) $$\gamma_{c}=1-\frac{\min(N_{\hat{v}},N_{\tilde{v}})}{N}.$$

The technical process of C-DTW is shown in Algorithm 2.
**Algorithm 2** C-DTW**Require:**sequence $\hat{v}={\left[{\hat{v}}_{m}\right]}_{1\times M}$sequence $\tilde{v}={\left[{\tilde{v}}_{n}\right]}_{1\times N}$**Ensure:**the DTW distance of $\hat{v}$ and $\tilde{v}$the optimal warping path: W**initialize:***M* = length($\hat{v}$)*N* = length($\tilde{v}$)DTW(1,1)=d(1,1)**for** m=1 to *M* **do**   **for** n=1 to *N* **do**     d(i,j) = $\sqrt{{\left({\hat{v}}_{m}-{\tilde{v}}_{n}\right)}^{2}}$;     execute (13) to obtain DTW(m,n) and $w_{t}\in W$   **end for****end for****for** i=1 to *T* **do**   count $N_{\hat{v}},N_{\tilde{v}}$**end for**execute (16)$C-\mathrm{DTW}\left(M,N\right)=\mathrm{DTW}\left(M,N\right)\times \gamma_{c}$**return:** C−DTW(M,N), W

## 4. Experiment

### 4.1. Experimental Setup

**Figure 4:**
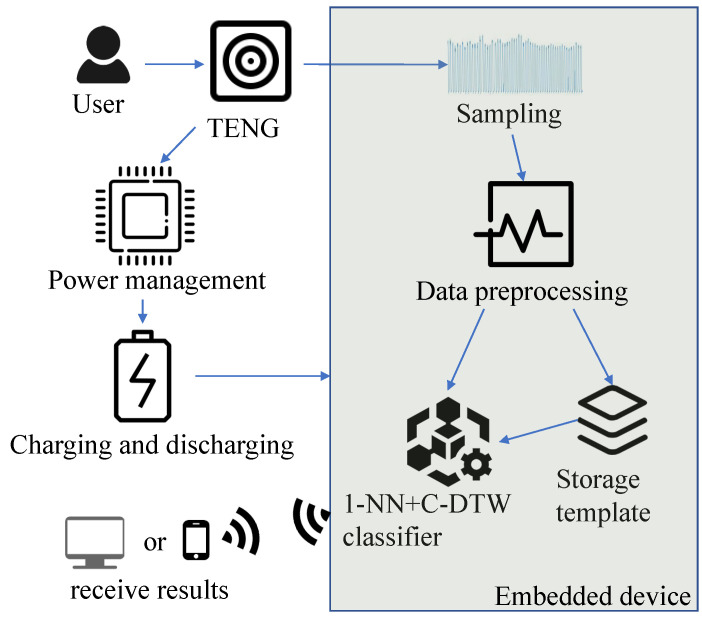
System block diagram.

**Figure 5:**
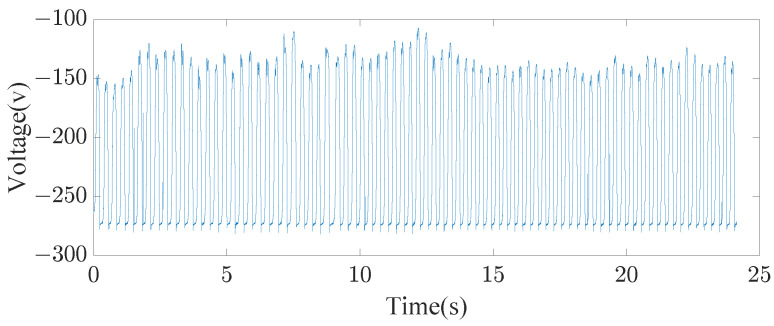
Original voltage sequence for 25 s of jumping.

During data acquisition, the TENG proposed in Section 2.2 was placed at heel, as shown in Figure 1. As shown in the Figure 4, the embedded device can recognize different actions. The energy of the TENG is stored in the battery through the power management circuit. The battery supplies power to the embedded device. Finally, the recognition result of the embedded device is transmitted to the computer or mobile phone through WiFi. In order to make the experiment more tractable, we split the data acquisition phase and the signal processing phase. In the data acquisition phase, the TENG electrode is connected to voltage detection equipment, as shown in the physical connection diagram part of Figure 1. Voltage data on activities is recorded on the embedded device with a sampling frequency of 1000 Hz. When reading actions from voltage data (running, jumping, or walking), the jumping height was randomized, as were the running and walking speeds. In order to obtain voltage data including only stable operation of the TENG, we removed the non-informative parts at the beginning and end of the collected data. For example, Figure 5 shows stable voltage data for the jumping action. Considering the requirement of real-time recognition, the sequence is intercepted by the sliding window. The window length is 2000 points, with a step size of 500 points. The duration of the intercepted sequence is 2 s. In the signal processing phase, we simulated C-DTW in Matlab2020a with an Intel(R) Core(TM) i5-6500 processor manufactured in Vietnam and Crucial 8GB DDR4-2133 RAM manufactured in Vietnam. The classification confusion matrix and computing time were used to evaluate the performance of Algorithm 2. In order to be practical, a WS-HAR system should be able to work with online data. This is inconsistent with leave-one-out cross-validation. Thus, the following experimental step was adopted: 200 sequences of each action were randomly selected from the preprocessed data for z-score standardization, then a small number (10 or 3) in each class were selected from 600 sequences as the template sequences of the 1-NN. Then, we classified the remaining sequences and drew the confusion matrix.

### 4.2. Data Preprocessing

**Figure 6:**
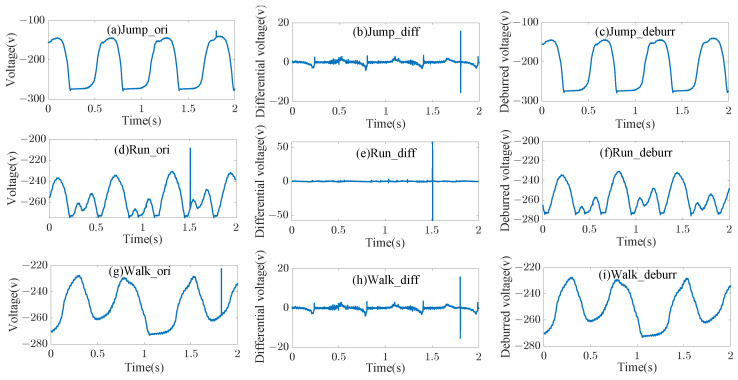
Intercepted jumping sequences (**a**,**d**,**g**), differential sequences (**b**,**e**,**h**), and processed sequences (**c**,**f**,**i**).

It can be seen from Figure 6a,d,g that discontinuities exist in the intercepted sequences due to inevitable environmental influences. Thus, it is necessary to eliminate these discontinuous points prior to classification. By drawing the derivatives of the intercepted sequences, it can been seen that the sequence derivative suddenly increases when discontinuous points appear. Therefore, it is easy to locate discontinuous points by setting a threshold for the sequence derivatives. After locating discontinuous points, we take the average of two points among the discontinuous points to replace it. Thus, assuming a discontinuous point appears at v(i), its replacement is represented by

(17) $$v\left(i\right)=\frac{v(i-1)+v(i+1)}{2}.$$

When a discontinuous point appears at the front or rear, it is replaced by its successor.

### 4.3. Influence of α Parameter

**Figure 7:**
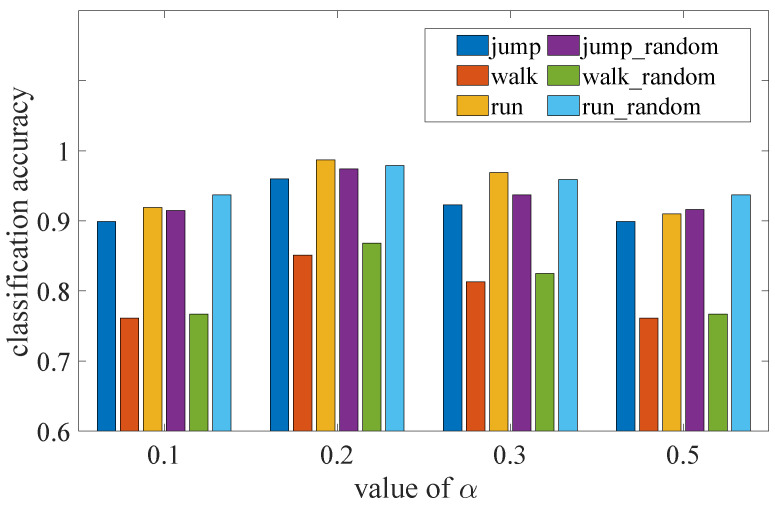
Classification accuracy when changing the value of α.

In this section, 200 sequences of each action were randomly selected for z-score standardization, then three sequences in each class were selected among 600 as the template for the 1-NN. To ensure the stability of the results, the whole process was repeated 40 times. We varied the α value used in C-DTW to investigate its influence on the classification accuracy. From Figure 7, it can be inferred that when α is too small a large number discontinuous points are ignored, which makes the compensation force deficient. This means that DTW alignments with similar shapes may not be selected, leading to potential inaccuracies in classification. When α is too large, too many unqualified points are included in S, resulting in excessive compensation force. To explain this more intuitively, in this condition DTW alignments with dissimilar shapes are more likely to be selected, contradicting the original intent of the compensatory mechanism based on alignment selection. Both of these two cases can invalidate the C-DTW proces. Classification accuracy shows an arch variation regularity with α, and the optimal α is 0.2.

From the experimental results, it can be seen that the optimal classification result for a granularity of 0.1 is obtained when α=0.2. Therefore, α was set to 0.2 in the subsequent experiments.

### 4.4. Method Comparison

**Figure 8:**
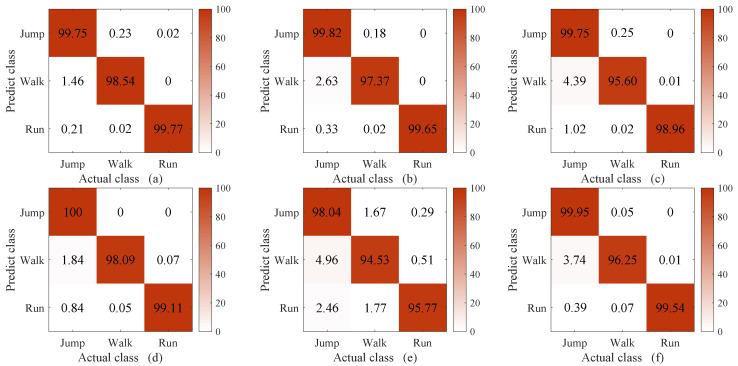
Confusion matrix of C-DTW (**a**), DTW (**b**), WDTW (**c**), DDTW (**d**), softDTW (**e**), and shapeDTW (**f**) when the number of templates of the 1-NN is 10.

**Figure 9:**
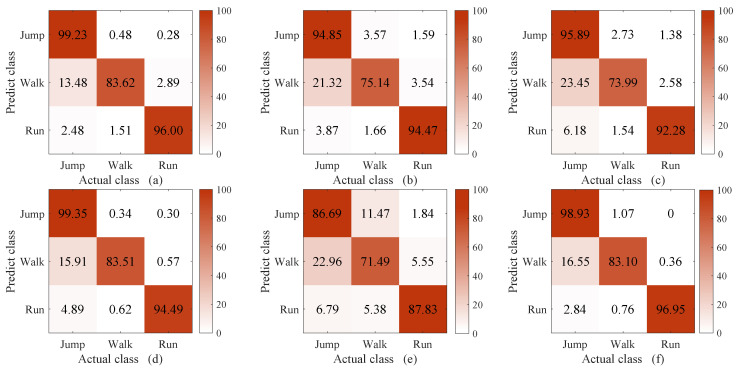
Confusion matrix of C-DTW (**a**), DTW (**b**), WDTW (**c**), DDTW (**d**), softDTW (**e**), and shapeDTW (**f**) when the number of templates of the 1-NN is 3.

**Figure 10:**
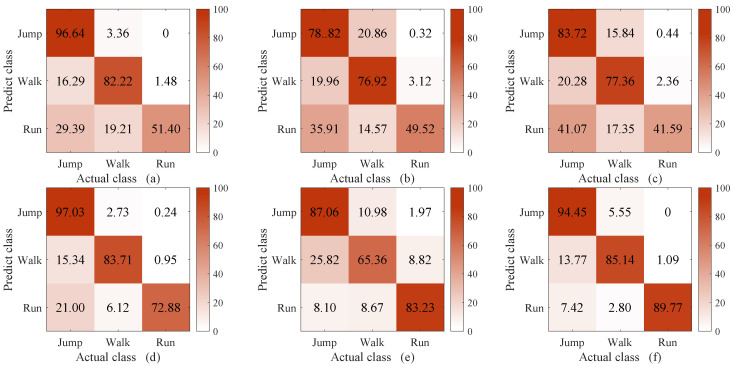
Confusion matrix of C-DTW (**a**), DTW (**b**), WDTW (**c**), DDTW (**d**), softDTW (**e**), and shapeDTW (**f**) when the length of the template sequence is 2000 points and the length of the classification sequence is 1500 points, with three template sequences per class.

The methods compared in this experiment were C-DTW + 1NN, DTW [39] + 1NN, WDTW [27] + 1NN, DDTW [28] + 1NN, and softDTW (i.e., soft-DTW [30]) + 1NN and shapeDTW [29] + 1NN. The reason for comparing our method with DTW, WDTW, DDTW, softDTW, and shapeDTW was that we conducted a thorough investigation into the methods that have been proposed to improve the classification ability of DTW. The methods compared in this experiment were chosen because they are the most recent. In shapeDTW, DTW is improved by considering similar shapes in the same class. Here, the sub-sequence length in shapeDTW was set to 100, as the pattern of the proposed WS-HAR system lasts about 100 points. The experimental results are shown in Figure 8, Figure 9 and Figure 10. Considering the unacceptable complexity when the number of templates exceeds 10, three templates were randomly selected from 200 for each class. The experiment was repeated 40 times. The times required to compute the distance between two 2000-point sequences using different DTW improvement methods are shown in Table 1.

From Figure 8, it can be seen that when the number of template sequences is large (ten in each class), the classification performance of DTW and its improved version are similar to that of C-DTW. When the number of template sequences is reduced to three, as shown in Figure 9, a significant improvement is observed in the classification accuracy of “walking” with C-DTW compared to DTW, with an increase of 8.48%. Compared to the classification accuracy of DTW, C-DTW shows an improvement of 4.8%, while WDTW decreases by 0.77%, DDTW improves by 4.3%, softDTW decreases by 6.15%, and shapeDTW improves by 4.84%. The C-DTW method achieves a 0.5% higher classification accuracy than DDTW with the same computation time. On the other hand, while shapeDTW achieves a 0.04% higher classification accuracy than C-DTW, the computation time increases from 0.32 s to 7.42 s. The improvement of C-DTW is due to the additional compensatory mechanism, which filters out parts with different shapes based on DTW alignment and retains only the parts with similar shapes. The resulting information is then fused into DTW through multiplication. DDTW (d) and shapeDTW (f) achieve similar results to C-DTW, as both methods incorporate shape information. DDTW transforms the original time series into high-level features containing shape information by using the differences between the sequences [40], while shapeDTW considers the shape correspondence of each sub-sequence using a sliding window [29]. On the other hand, WDTW (c) and softDTW (e) do not take shape features into account. Moreover, the window constraints of WDTW [41] and the softmin [42] operation in softDTW cause information loss, resulting in slightly lower classification performance compared to the other methods.

According to Figure 10, when the length of the classification sequence is 1500 and the length of the template sequence is 2000, it can be observed that C-DTW benefits from the compensatory mechanism based on alignment selection and achieves higher classification performance than DTW. However, it falls short of the performance achieved by DDTW and shapeDTW. The reason for this lies in the inherent limitations of DTW, which stem from the features it considers. DTW only takes into account the y-axis values of datapoints, and does not effectively handle their shapes [40]. Thus, even with the selection of DTW alignment it is not possible to achieve better results on the basis of alignments that inherently do not take shape relationships into account. Furthermore, it can be observed that both DDTW and shapeDTW show gaps in the “running” classification. This is because the classification sequences are truncated in these approaches compared to the template sequences, making their shapes more incomplete. However, shapeDTW, which uses sliding windows to handle the shape sub-sequences of the entire sequence, is less affected by shape incompleteness.

**Figure 11:**
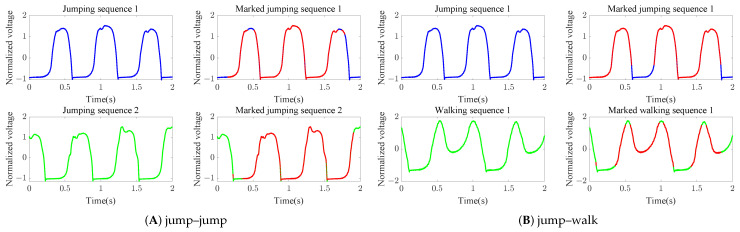
Original signal and marked signal. The two subfigures on the left of (**A**,**B**) show the original “jumping” and “walking” signals, while the red points marked in the two subfigures on the right of (**A**,**B**) show the points selected according to the compensatory mechanism.

Below, we provide an analysis of the compensatory mechanism proposed in this paper and how it is able to achieve better classification performance. In Figure 11A, the blue and green lines represent two untreated jumping sequences. The red parts represent points which satisfy (14) under the DTW alignment. We utilize $min(N_{\hat{v}},\tilde{v})$ to calculate the compensation weight according to (15). After applying traditional DTW, it can be seen that the DTW value between two jumping sequences is 864.82 (the left side in Figure 11A), while the DTW value between jumping and walking is 651.38 (the left side in Figure 11B). According to the compensatory mechanism, points that satisfy (14) are found, then $\gamma_{c}$ is calculated for compensation according to (16). The compensatory mechanism proposed in this paper is based on the notion that if two sequences belong to the same class, the number of points that satisfy (14) under DTW alignment will occupy a relatively larger proportion. Conversely, for different class of sequences the number of points that satisfy (14) under DTW alignment occupies a relatively smaller proportion. After compensation, the C-DTW value between the two jumping sequences is 0.432, while the C-DTW value between jumping and walking is 461.18. Therefore, C-DTW substantially reduces the distance between sequences of the same category and slightly reduces the distance between sequences among different categories.

**Figure 12:**
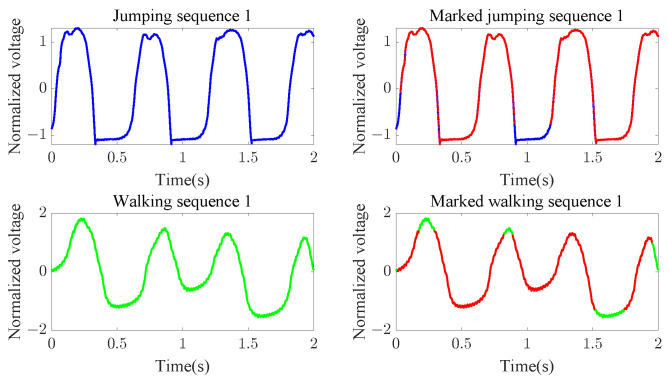
The “jumping” and “walking” sequences have similar beginning and ending shapes. The two subfigures on the left show the original “jumping” and “walking” signals, while the red points marked in the two subfigures on the right show the points selected according to the compensatory mechanism.

Next, we discuss the problem of why the “walking” classification shows relatively lower performance in our experiments. Figure 12 shows that when “jumping” and “walking” have similar starting and ending shapes in their sequences, alignment of points using DTW cannot perfectly resolve cases in which dissimilar shapes are aligned, even after filtering based on DTW correspondence. This is due to the inherent limitations of DTW correspondence, in that it only considers the y-axis values of datapoints and cannot effectively handle their shapes. Notably, instances where “jumping” and “walking” sequences have similar starting and ending shapes and occur separately in the template and classification sequences are not very common. Therefore, even though the classification accuracy is not as high for “walking” as it is “jumping” and “running”, it does not significantly lag behind.

In terms of time complexity, as shown in Table 1, C-DTW exhibits similar computation time to WDTW, DDTW, and softDTW, taking 0.31 s, which is only a slight increase compared to the DTW algorithm (0.7 s). It is significantly faster than shapeDTW, which takes 7.41 s to compute. According to [43], the time complexity of DTW is O(MN), where M and N are the lengths of the two sequences. When the lengths of both sequences are the same, the time complexity becomes $O(N^{2})$. Considering to (14) and (15) along with Algorithm 2, the compensatory mechanism proposed in this paper involves only one loop during the calculation to traverse all DTW correspondences. Hence, the overall time complexity is O(MN+S), where max{M,N}≤S≤M+N. It can be observed that the proposed method in this paper does not increase the time complexity magnitude, and the actual execution time aligns with the results shown in Table 1. Generally speaking, C-DTW achieves excellent performance for the proposed TENG-based WS-HAR system.

### 4.5. Compensation of Contrasted DTW

In this section, we prove that the proposed C-DTW compensatory method can elevate the performance of the contrasted DTW method with α=0.2 in our WS-HAR system. First, 200 sequences of each action were randomly selected from preprocessed data for z-score standardization. Then, three sequences in each class were randomly selected from 600 as the template sequences of the 1-NN, simulating the situation of online data processing. For stability, the whole process from data selection to classification was repeated 40 times. The tested methods were compensatory DDTW (C-DDTW) and compensatory shapeDTW (C-shapeDTW). The reasons for not selecting WDTW and softDTW were as follows:

(1) When WDTW calculates the warping path, the distance matrix varies. This is not consistent with DTW. Thus, if the compensatory method were imposed, the threshold based on two matched points would be meaningless.
(2) The minimum obtained by SoftDTW is calculated according to (13) without selection, resulting in invalid warping paths between the two sequences. Thus, the concept of C-DTW is not applicable in this case.

**Figure 13:**
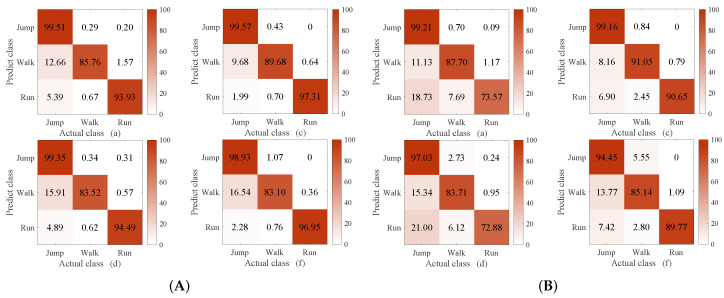
The compensatory mechanism proposed in this paper combined with different versions of DTW: confusion matrices of (**A**) C-DDTW (a), C-shapeDTW (c), DDTW (d), and shapeDTW (f) when the number of 1-NN templates is 3 and of (**B**) C-DDTW (a), C-shapeDTW (c), DDTW (d), and shapeDTW (f) with a template sequence length of 2000 points, classification sequence length of 1500 points, and three template sequences per class.

From Figure 13, it can be seen that applying the compensatory mechanism to DDTW and shapeDTW can improve their classification accuracy. Specifically, under the condition of equal sequence lengths (Figure 13A), C-DDTW shows a 0.62% improvement in classification accuracy compared to DDTW after applying the compensatory mechanism, while C-shapeDTW achieves a 2.53% improvement compared to shapeDTW. In Figure 13B, C-DDTW exhibits a 2.28% improvement over DDTW, while C-shapeDTW shows a 3.84% improvement over shapeDTW. It is worth noting that applying the compensatory mechanism proposed in this paper results in improved classification accuracy with no increase in time complexity. This is because shapeDTW focuses on the shape relationship of sub-sequences through a sliding window approach. Thus, with better shape correspondence, the compensatory mechanism based on the selected correspondence in this paper can further emphasize the shape of the sequences. When the lengths of the template and classification sequences are different (Figure 13B), it can be observed that after applying the compensatory mechanism both DDTW and shapeDTW achieved significant improvements in classification accuracy. Additionally, it is notable that DDTW and shapeDTW demonstrate a difference in the “running” classification. This disparity arises because the classified sequences have been truncated in comparison to the template sequences, resulting in their shapes being more incomplete. However, shapeDTW, which employs sliding windows to mitigate the impact of error shape alignment caused by shape incompleteness of sub-sequences within the entire sequence, is less affected by significant shape incompleteness.

### 4.6. Classification of UCR

Researchers from the University of California, Riverside have created the UCR Archive [44], which contains sixteen datasets for use in time series classification. Over time, the archive has been updated and expanded to include various time series types, such as “Device”, “ECG”, “Image”, “Sensor”, “Motion”, and more. The archive now consists of 128 datasets, with several datasets being converted into time series data by different methods. For example, in the “Image” dataset, the original image data are transformed into a set of contour points that are then represented as time series data to form the dataset. Each dataset in the UCR Archive consists of 2–128 categories. For this paper, datasets with the “Sensor” and “Motion” types were selected to test the proposed method utilizing a nanogenerator as a sensor for human motion recognition. A total of 44 categories were chosen from the UCR Archive; Table 2 displays the essential information about the samples and the algorithm execution results. In this experiment, the α value for the proposed C-DTW was set to 0.01. Table 2 presents the results of C-DTW+1NN and DTW+1NN on “Sensor” and “Motion” datasets from the UCR Archive. The “length” column represents the number of datapoints in each sequence. The “vary” column indicates that the sequence lengths are not fixed for this type of dataset. The “class” column indicates the number of categories required for classification in each dataset; for example, the “Car” dataset requires classification into four categories. The last two columns indicate the error rate of classification using C-DTW+1NN and DTW+1NN. A smaller error rate indicates greater accuracy in classification.

Table 2 illustrates that in most cases the proposed C-DTW method outperforms DTW in terms of classification accuracy, even for sequences without a certain periodicity. An α value of 0.01 was used in this experiment, as the shape differences between the sequences were small. The idea behind C-DTW is that the same types of sequence have the same shapes, meaning that under DTW alignment those parts of the sequence with points that are close in distance account for a larger proportion. Conversely, the proportion of similar shapes between different data types is shorter. For the “DodgerLoopDay” dataset in Table 2, the C-DTW+1NN error rate is 0.5876 while the DTW+1NN classification error rate is 0.475.

**Figure 14:**
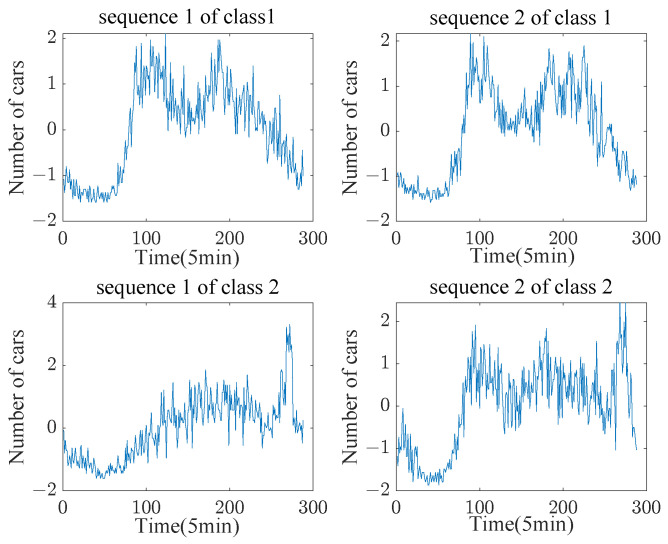
The DogersLoopGame dataset in the UCR Archive contains two types of data, representing traffic data for Class 1: Normal Day and Class 2: Game Day. The vertical axis represents the number of cars in the past 5 min and the horizontal axis represents the number of five minute periods.

In the case of the “DodgerLoopGame” dataset, the classification error rate of C-DTW+1NN is 0.21014, while the classification error rate of DTW+1NN is 0.14493. Although the classification performance is not very good, setting the α value of C-DTW to 0.001 results in better classification performance, with error rates of 0.475 and 0.13768 for the “DodgerLoopDay” and “DodgerLoopGame” datasets, respectively. Figure 14 displays the plotted sequences of the “DodgerLoopGame” dataset to help explain the reason for the improved classification results.

## 5. Conclusions

This paper provides a framework and feasibility validation for subsequent realization of TENGs. A WS-HAR system is realized by placing a TENG on the heel of the shoe mat. The TENG can realize the potential of a self-powering device. Compared with video-based HAR, the method proposed in this paper is not limited by application scenario. Moreover, compared with sensor-based HAR, the proposed method avoids the need to wear a cumbersome device. Additionally, the C-DTW method proposed as a compensatory mechanism for the WS-HAR system has high accuracy, low time complexity, and can be easily combined with other DTW versions. Our experiments show that C-DTW has stronger robustness in the case of quasi-periodic sequences. In the future, self-powering TENG devices can be used with a system such as the one proposed here to collect plantar piezoelectric data and identify complex action patterns.

## Figures and Tables

**Table 1:** Time required to classify each action with three template sequences in each class.

Data	C-DTW	DTW [39]	WDTW [27]	DDTW [28]	softDTW [30]	shapeDTW [29]
jump	0.3288	0.2388	0.3282	0.2974	0.3225	7.4383
run	0.3162	0.2451	0.3143	0.3133	0.3178	7.4146
walk	0.3157	0.2474	0.3089	0.3046	0.3014	7.4297
jump_random	0.3245	0.2322	0.2998	0.2807	0.3049	7.3968
run_random	0.3267	0.2482	0.3014	0.2899	0.3201	7.4587
walk_random	0.3117	0.2311	0.3169	0.3022	0.3138	7.4444

**Table 2:** Classification error rate results on “Sensor” and “Motion” datasets from the UCR Archive.

Type	Name	Length	Class	C-DTW	DTW
Sensor	Car	577	4	0.23333	0.25
Sensor	ChlorineConcentration	166	3	0.37917	0.37344
Sensor	CinCECGTorso	1639	4	0.2833	0.30942
Sensor	DodgerLoopDay	288	7	0.587	0.475
Sensor	DodgerLoopGame	288	2	0.13768	0.14493
Sensor	DodgerLoopWeekend	288	2	0.028986	0.028986
Sensor	Earthquakes	512	2	0.30216	0.33094
Sensor	FordA	500	2	0.4303	0.42879
Sensor	FordB	500	2	0.39506	0.39383
Sensor	FreezerRegularTrain	301	2	0.10105	0.093
Sensor	FreezerSmallTrain	301	2	0.2786	0.28
Sensor	GesturePebbleZ1	Vary	6	0.1162	0.098837
Sensor	GesturePebbleZ2	Vary	6	0.22785	0.21519
Sensor	InsectWingbeatSound	256	11	0.56667	0.56919
Sensor	ItalyPowerDemand	24	2	0.054422	0.054422
Sensor	Lightning2	637	2	0.19672	0.19672
Sensor	Lightning7	319	7	0.023288	0.023288
Sensor	MoteStrain	84	2	0.10623	0.10942
Sensor	Phoneme	1024	39	0.72996	0.7714
Sensor	PickupGestureWiimoteZ	Vary	10	0.4	0.38
Sensor	Plane	144	7	0	0
Sensor	ShakeGestureWiimoteZ	Vary	10	0.16	0.16
Sensor	SonyAIBORobotSurface1	70	2	0.2945	0.28785
Sensor	SonyAIBORobotSurface2	65	2	0.15635	0.1574
Sensor	StarLightCurves	1024	3	0.12065	0.11462
Sensor	Trace	275	4	0.01	0.01
Sensor	Wafer	152	2	0.1476	0.016061
Motion	CricketX	390	12	0.2281	0.2281
Motion	CricketY	390	12	0.25897	0.25128
Motion	CricketZ	390	12	0.21795	0.21282
Motion	GunPoint	150	2	0.12667	0.12667
Motion	GunPointAgeSpan	316	2	0.08544	0.08543
Motion	GunPointMaleVersusFemale	316	2	0.0031646	0.0031646
Motion	GunPointOldVersusYoung	315	2	0.15556	0.1587
Motion	Haptics	308	5	0.63312	0.63636
Motion	InlineSkate	550	7	0.62364	0.62545
Motion	ToeSegmentation1	228	2	0.2193	0.20175
Motion	ToeSegmentation2	130	2	0.14615	0.15385
Motion	UWaveGestureLibraryAll	3582	8	0.079564	0.083752
Motion	UWaveGestureLibraryX	3582	8	0.26801	0.26912
Motion	UWaveGestureLibraryY	3582	8	0.35204	0.35539
Motion	UWaveGestureLibraryZ	3582	8	0.34841	0.35539
Motion	Worms	77	5	0.46753	0.48052
Motion	WormsTwoClass	77	2	0.36364	0.36364

## Data Availability

Not applicable.
